# Complete Genome Sequence of the Probiotic Strain *Lacticaseibacillus rhamnosus* VHProbi M14, Isolated from Cheese

**DOI:** 10.1128/mra.00826-22

**Published:** 2022-10-13

**Authors:** Hongchang Cui, Qian Wang, Zhi Duan

**Affiliations:** a Qingdao Vland Biotech Group Co., Ltd., Qingdao, China; Indiana University, Bloomington

## Abstract

Lacticaseibacillus rhamnosus VHProbi M14 is a proprietary probiotic strain that was isolated from cheese. Here, we investigate the whole-genome sequence of this bacterium. Its whole genome only contains a chromosome without a plasmid.

## ANNOUNCEMENT

Cheese purchased from the local supermarket in 2020 was homogenized in a NaCl (0.85%) solution. The dilution was spread onto de Man-Rogosa-Sharpe agar plates and incubated under anaerobic conditions at 37°C for 48 h to enrich bacteria. A strain designated VHProbi M14 was picked up and identified as the species Lacticaseibacillus rhamnosus using 16S rRNA sequencing as described by Liu (1). A combined strategy using the Illumina Hiseq 2500 and PacBio RS II single-molecule real-time (SMRT) sequencing platforms was used to sequence the genome of this strain. The strain was cultured for DNA extraction using the same culture conditions as for enrichment. Total DNA was extracted using the genomic DNA purification kit (Promega, USA) and used both for the Illumina and the PacBio library preparation. Template DNA was sheared into 400-bp fragments for the Illumina library using the TruSeq library preparation kit. The prepared libraries were then used for paired-end Illumina sequencing (2 × 150 bp). For PacBio sequencing, DNA was spun in Covaris g-Tubes (Covaris, MA) to be sheared into ~10-kb fragments. Then, the fragments were purified, end repaired, ligated with the SMRTbell adapters using the SMRTbell Express template prep kit 2.0 (Pacific Biosciences, CA), and sequenced on one SMRT cell following the manufacturer’s instructions. Finally, 6,925,262 raw reads and 6,828,422 clean reads were generated using the Illumina sequencing platform and 72,937 raw reads (*N*_50_, 233,237 bp) were generated using the PacBio sequencing platform. The Illumina raw reads were quality controlled by using Sickle version 1.33 (https://github.com/najoshi/sickle). The PacBio raw reads were assembled into a scaffold using Unicycler version 0.4.8 (2). Error correction of the assembly result was performed by using Pilon version 1.22 using the Illumina reads (3). The last circular step was checked and finished manually to generate seamless chromosome and plasmid sequences. If there is an overlap at both ends of the scaffold to reach 5,000 bp, one end of the overlap is cut off and the sequence looped. The final assembly generated a complete genome sequence with a single chromosome and no plasmid. Read coverage for the assembly was calculated to be 360.79×. Glimmer version 3.02 (4), tRNA-scan-SE version 2.0 (5), and barrnap version 0.9 were used to predict the protein-coding genes, tRNA, and rRNA, respectively (https://github.com/tseemann/barrnap/). CheckM version 1.1.3 was used to check the quality of the assembled genome sequence, with a completeness of 99.48%, contamination of 0%, and strain heterogeneity of 0% (6). Genome annotation was performed using the Prokaryotic Genome Annotation Pipeline (PGAP) version 6.2 (7). PGAP determines structural annotation by comparing open reading frames (ORFs) to libraries of protein hidden Markov models (HMMs), representative RefSeq proteins, etc. GeneMarkS-2+ then makes *ab initio* coding region predictions for genomic regions that lack HMM or protein evidence and selects start sites for ORFs whose evidence comes from HMMs. Default parameters were used for all software unless otherwise specified.

The whole-genome sequence of L. rhamnosus VHProbi M14 contained a 2,898,403-bp circular chromosome with a GC content of 46.68%. There were 2,622 protein-coding genes, 15 rRNA genes, 60 tRNA genes, 3 noncoding RNA (ncRNA) genes, and 20 pseudogenes in the genome.

### Data availability.

The 16S rRNA sequence and the whole-genome sequence of the L. rhamnosus VHProbi M14 have been deposited in the database with GenBank accession numbers CP095384 and OP159920, SRA accession number SRR20852278 and SRR20852277, BioProject accession number PRJNA824241, and BioSample accession number SAMN27397804.
